# Characterizing the secretome of EGFR mutant lung adenocarcinoma

**DOI:** 10.3389/fonc.2023.1286821

**Published:** 2024-01-08

**Authors:** Jennifer K. Luu, Fraser D. Johnson, Jana Jajarmi, Tianna Sihota, Rocky Shi, Daniel Lu, Dylan Farnsworth, Sandra E. Spencer, Gian Luca Negri, Gregg B. Morin, William W. Lockwood

**Affiliations:** ^1^ Department of Integrative Oncology, British Columbia (BC), Cancer Research Institute, Vancouver, BC, Canada; ^2^ Department of Pathology and Laboratory Medicine, University of British Columbia, Vancouver, BC, Canada; ^3^ Interdisciplinary Oncology Program, University of British Columbia (UBC), Vancouver, BC, Canada; ^4^ Michael Smith Genome Sciences Centre, BC Cancer Research Institute, Vancouver, BC, Canada; ^5^ Department of Medical Genetics, University of British Columbia, Vancouver, BC, Canada

**Keywords:** lung cancer, *EGFR*, secretome, transformation, early detection

## Abstract

**Background:**

Lung cancer is the leading cause of cancer related death worldwide, mainly due to the late stage of disease at the time of diagnosis. Non-invasive biomarkers are needed to supplement existing screening methods to enable earlier detection and increased patient survival. This is critical to *EGFR*-driven lung adenocarcinoma as it commonly occurs in individuals who have never smoked and do not qualify for current screening protocols.

**Methods:**

In this study, we performed mass spectrometry analysis of the secretome of cultured lung cells representing different stages of mutant *EGFR* driven transformation, from normal to fully malignant. Identified secreted proteins specific to the malignant state were validated using orthogonal methods and their clinical activity assessed in lung adenocarcinoma patient cohorts.

**Results:**

We quantified 1020 secreted proteins, which were compared for differential expression between stages of transformation. We validated differentially expressed proteins at the transcriptional level in clinical tumor specimens, association with patient survival, and absolute concentration to yield three biomarker candidates: MDK, GDF15, and SPINT2. These candidates were validated using ELISA and increased levels were associated with poor patient survival specifically in EGFR mutant lung adenocarcinoma patients.

**Conclusions:**

Our study provides insight into changes in secreted proteins during *EGFR* driven lung adenocarcinoma transformation that may play a role in the processes that promote tumor progression. The specific candidates identified can harnessed for biomarker use to identify high risk individuals for early detection screening programs and disease management for this molecular subgroup of lung adenocarcinoma patients.

## Background

Lung cancer is the leading cause of cancer mortality in men and women worldwide, contributing to 1.8 million deaths in 2020 alone (1). Lung cancer consists of two subtypes, small cell lung cancer and non-small cell lung cancer (NSCLC), which comprise 15% and 85% of cases, respectively (2). The most common NSCLC subtype is lung adenocarcinoma (LUAD), which comprises ~60% of NSCLC cases (3). LUAD can be classified into oncogenic driver subgroups, where mutations in *KRAS* and *EGFR* are common (4). *KRAS* mutations are associated with smoking; in contrast, *EGFR* mutations are associated with never smokers, especially in women and in East Asia (5, 6). LUAD is thought to arise from a stepwise process of genetic and epigenetic changes, which begins with histologically normal epithelial cells and ends with invasive carcinoma (7, 8). The majority of *in vitro* studies aiming to investigate the genetic alterations required to enable transformation have centered on *KRAS*-driven LUAD, with limited investigation into *EGFR*-driven LUAD (9, 10).

Lung cancer, including LUAD, is typically diagnosed in late or metastatic stages; in these stages, long term patient survival is limited due to less effective treatment methods available such as chemotherapy and radiotherapy (2, 11). Prognosis and clinical stage are directly related, incentivizing earlier lung cancer detection for improvements to patient survival (12). Patients diagnosed at stage I have a five-year survival rate of 68.4%, in contrast with those diagnosed at stage IV, where the five-year survival rate is 5.8% (13, 14). Lose dose computed tomography (LDCT), a radiographic scanning technique used to image the lungs, is the standard for lung cancer screening (15). LDCT was initially demonstrated as an effective annual screening technique for high-risk individuals in the National Lung Screening Trial (NLST), where there was a 20% reduction in lung cancer mortality (16, 17). However, LDCT is limited by non-specificity, over diagnosis of benign pulmonary nodules, and potential harms of repeated radiation (16, 18). Furthermore, LDCT screening is not universally applicable to all populations susceptible to cancer; never smokers were not included in the NSLT study and its effectiveness for this group is unclear (19, 20). One proposed strategy to supplement LDCT for better screening practices is the use of biomarkers of early cancer development (21). Biomarkers would complement LDCT by reducing screening costs through criteria refinement, supporting clinical decision making in unclear situations such as indeterminate pulmonary nodules, and personalized patient screening and treatment planning (21). Blood biomarkers are of particular interest, due to their capability for inexpensive and relatively non-invasive collection (22). However, there are few confirmed protein biomarkers for LUAD and current candidates including NSE, proGRP, CEA, SCCA, and CYRFA 21-1 are non-specific for lung cancer and cannot be used to distinguish histology nor molecular subtype (23).

The secretome consists of proteins transported from a cell into the extracellular space and it is estimated to comprise 15% of all human proteins (24). Secretome proteins include cytokines, growth factors, extracellular matrix-degrading proteinases, and cell motility factors involved in local and systemic signaling (24, 25). A regulated secretome is important in maintaining homeostasis and changes in secretome protein abundance have been implicated in cancer (26). Tumor cells can release proteins that can affect functions such as angiogenesis, immunomodulation, basement membrane degradation, and extracellular matrix modeling (27). Secreted proteins enter bodily fluids such as blood and urine, which enables non-invasive collection and potential biomarker analysis (25, 28). A recent study of 32 types of primary tumors and normal-adjacent tissues found that proteins often found in the secretome are altered at the transcriptional level specifically in cancer, and found common expression decreases of proteins implicated in functions including adhesion and tumor suppression (29). These findings highlight the broad scope changes in the secretome during tumor progression and metastasis; in addition, this study showed the potential of the secretome as a reservoir of biomarker candidates such as matrix metalloprotease (MMP) family members, including MMP9 in breast and lung cancer (29–31). In NSCLC, recent secretome studies have identified proteins affecting erlotinib resistance, biomarkers for cisplatin response, and metastasis (32–34). However, secretome studies often profile immortalized cell lines, where cells are established from patient tumors and have already undergone malignant transformation (35, 36). Studying changes in secreted proteins that occur during the different steps of cancer progression from normal epithelium to invasive and metastatic cancer may therefore generate potential biomarker candidates to aid in early detection, diagnosis and prognosis. This is urgently needed in *EGFR*-driven LUAD, as the transformation process has not been fully elucidated and there are no concrete screening guidelines for the never smoker demographic where mutant *EGFR* LUAD cases are enriched (20, 37).

In this study, we investigated changes in the secretome during malignant transformation and identified potential biomarker candidates by performing proteomic analysis using an *in vitro* model of mutant *EGFR* driven transformation. We generated cell lines modeling the stepwise genetic alterations that occur during transformation, using non-transformed human bronchial epithelial cells (HBEC), and compared these against established LUAD cell lines to profile differences between stages of transformation (38). We initially identified 1020 secretome proteins and progressed through a series of groupwise and individual cell line comparisons to uncover 499 differentially expressed proteins between the untransformed and transformed states. Key selected proteins were validated with gene expression and patient survival data, to determine five biomarker candidates including MDK, GDF15, and SPINT2. This provides the first description of secretome changes during mutant *EGFR*-driven LUAD transformation and provides insight into the biological processes that can be applied for biomarker development.

## Methods

### Cell culture

All cell lines used were obtained from the American Type Culture Collection (ATCC) or gifted from Dr. Adi Gazdar (UT Southwestern Medical Center). PC-9, H1975 (NCI-H1975), HCC4006, HCC4011, H3255 (NCI-H3255) were cultured in RPMI-1640 (Thermo Fisher Scientific), supplemented with 10% fetal bovine serum (FBS; Thermo Fisher Scientific) and 1% Penicillin-Streptomycin (Thermo Fisher Scientific). HBEC (HBEC3-KT) cells were cultured in Keratinocyte serum-free medium (KSFM; Thermo Fisher Scientific), supplemented with accompanying bovine pituitary extract (BPE; Thermo Fisher Scientific), human recombinant epidermal growth factor (EGF; Thermo Fisher Scientific), and 1% Pencillin-Streptomycin. All cell lines were cultured at 37°C, in 5% CO_2_.

### Expression constructs and cell line generation

Lentiviral vector and overexpression plasmids used to construct overexpression constructs for *EGFR L858R* (Plasmids #82906, #17451) and *GFP* (Plasmid #17445) were obtained from Addgene. Retroviral *TP53* c-terminal fragment (CT) overexpression construct and pCX4 hisD vector control were gifted from Dr. Romel Somwar (Memorial Sloan Kettering Cancer Centre, NY). Lentivirus was produced using HEK 293TD cells (ATCC), psPAX2 (Plasmid #12260; Addgene) and pMD2.G (Plasmid #12259; Addgene). Retrovirus was produced using Phoenix-AMPHO cells (ATCC). HBEC cell lines expressing *GFP, EGFR L858R*, with *TP53* C-terminal (CT) domain dominant negative mutations were generated by lentiviral and retroviral infection, and selected with 5µg/mL blasticidin (Thermo Fisher Scientific), and 2mg/mL L-histidinol (Thermo Fisher Scientific). The HBEC cell line expressing *EGFR L858R* and *TP53* CT was additionally selected in 10µM Nutlin-3a for 6 days (SelleckChem).

### Western blot analysis

Protein from cell lysates were obtained by rinsing cells with cold Dulbecco’s phosphate-buffered saline (DPBS) (Thermo Fisher Scientific) and lysed in RIPA buffer (VWR) with Halt protease and phosphatase inhibitor cocktail (Thermo Fisher Scientific). Samples were collected on ice, vortexed, and frozen at -80°C before being sonicated and centrifuge-separated at 15,000xg, 4°C for 10 minutes. Protein concentrations were detected using a Pierce BCA protein assay kit (Thermo Fisher Scientific), then samples were heated in 1x diluted NuPAGE LDS sample buffer (Thermo Fisher Scientific) containing 1:10 diluted 2-Mercaptoethanol (MilliporeSigma) at 75°C, for 10 minutes. 20-25µg of samples were run on NuPAGE 4-12% Bis-Tris protein gels (Thermo Fisher Scientific) in NuPAGE MOPS SDS running buffer (Thermo Fisher Scientific) at 200V, for 50 minutes. Samples were transferred from Bis-Tris gel to Immobilon-P PVDF (MilliporeSigma) either at 70V, 4°C, for 2 hours or 30V, 4°C overnight. Membranes were incubated in TBS-T (0.1% Tween-20) (TBS, Bio-Rad; Tween-20, Thermo Fisher Scientific) containing 5% BSA (MilliporeSigma) until primary antibody incubation.

Primary antibodies were prepared following manufacturer’s instructions in TBS-T containing 5% BSA or 5% milk (MKP3); specific dilutions are noted. The following primary antibodies were used: p-ERK1/2(Thr202/Tyr204) (p-p44/42 (Thr202/Tyr204); Cell Signaling Technology, 9101); ERK1/2(p44/p42; Cell Signaling Technology, 4695); p-MEK1/2(Ser217/221) (Cell Signaling Technology, 9121); MEK1/2 (Cell Signaling Technology, 9122); p-EGFR(Tyr1068) (Cell Signaling Technology, 2234); EGFR L858R (Cell Signaling Technology, 3197); EGFR (Cell Signaling Technology, 2232); MKP3 (DUSP6) (Santa Cruz Biotechnology, sc-377070, 1:200); p53(Cell Signaling Technology, 2527); p53, to detect TP53 CT (MilliporeSigma, SAB4503011); GFP (Cell Signaling Technology, 2956); β-Actin (Cell Signaling Technology, 12620, 1:2000). Membranes were incubated in primary antibodies at 4°C overnight, then HRP-conjugated secondary antibodies according to manufacturer recommendations (Cell Signaling Technology). Proteins were detected after incubation with ECL, SuperSignal West Pico Plus Chemiluminescent Substrate (Thermo Fisher Scientific) or SuperSignal West Femto Maximum Sensitivity Substrate (Thermo Fisher Scientific) on a ChemiDoc MP imager (Bio-Rad).

### Secretome sample collection

Cells were seeded at approximately 80% confluency in 6cm plates in triplicate overnight: 900,000 (HBEC); 1,000,000 (PC-9); 1,000,000 (H1975); 3,500,000 (HCC4006); 7,000,000 (HCC4011); 2,500,000 (H3255). Plates were rinsed twice with DPBS and media was changed to supplement-free KSFM containing 1% Penicillin-Streptomycin (HBEC) or serum-free RPMI-1640 containing 1% Pencillin-Streptomycin (PC-9, H1975, HCC4006, HCC4011, H3255). Plates containing only media were also prepared, and all plates were incubated for 24 hours at 37°C. Conditioned media was collected, centrifuged at 1000 RPM for 5 minutes, at 4°C, and filtered with a 0.45μM filter (Sarstedt) to remove cell debris. The complete 4mL volume of filtered conditioned media was centrifuged in a Vivaspin Turbo 3kDa ultrafiltration unit at 3220xg, at 8°C until media was concentrated to approximately 150-200μL. Concentrated media was buffer exchanged, where samples were centrifuged twice with 4mL 50mM HEPES buffer, pH 7.0, then once with 1mL HEPES at 3220xg, at 8°C to a final volume of 150-300uL. Samples were stored at -80°C until mass spectrometry sample preparation.

### Proteomic analysis

Samples were prepared for tandem mass spectrometry (MS/MS) analysis through a protocol of reduction, alkylation, and protein digestion. Samples were reduced by incubating with 16μL of 200 mM dithiothreitol (DTT, Bio-Rad) for 30 minutes at 55°C, then alkylated by incubating with 32 μL of 400 mM iodoacetamide (IAA, Bio-Rad) for 30 minutes at room temperature. Samples were quenched with an additional 16 μL 200 mM DTT. Trypsin/Lys-C mix was prepared for sample digestion, where 200μL of 200 mM HEPES pH 8.0 was added to 20 μg Trypsin/Lys-C (Promega). Samples were digested by incubating with 16μL Trypsin/Lys-C mix on a ThermoMixer (Eppendorf) at 1000 RPM, overnight at 37°C. One tenth of each sample was pooled and prepared to confirm quality. Peptides were acidified by adding 10% (v/v) trifluoroacetic acid (TFA, Thermo Fisher Scientific) and diluted to a concentration of 1% TFA, then desalted following a Stop And Go Extraction (STAGE) tip protocol (39). Briefly, STAGE tips were packed with 3 punches of C18 resin which was washed (100 uL 0.1% TFA in acetonitrile) and equilibrated (2x100 uL 0.1% TFA in 18 MΩ water) then peptide was loaded. Salts were removed by rinsing (200 uL 0.1% formic acid in HPLC water) then eluted in 100 uL 0.1% formic acid in 60/40 acetonitrile/HPLC water. Desalted peptides were eluted and solvent evaporated by centrifuging samples in a SpeedVac Vacuum Concentrator (Thermo Fisher Scientific) until dry. Peptides were reconstituted in a 0.1% formic acid, 1% DMSO aqueous solution and assessed for quality on a LTQ Orbitrap Velos™ (Thermo Fisher Scientific).

The remaining digested peptides were tandem mass tag (TMT) labeled using a TMT 11-plex kit (Thermo Fisher Scientific) following manufacturer’s instructions. Post-labeling, samples were pooled, dried by speed vacuum to evaporate excess solvent, and acidified with TFA as described above. Peptides were desalted following the STAGE tip protocol and excess solvent was reduced by vacuum centrifugation (39). Peptides were constituted in a 0.1% formic acid, 1% DMSO aqueous solution and run on an Orbitrap Eclipse™ mass spectrometer (Thermo Fisher Scientific) set to MS2 mode. MS spectra were searched with Proteome Discoverer suite (v.2.4.0.305, Thermo Fisher Scientific) against Swissprot human reference database (20585 sequences, October 2020). Precursor and fragment ion tolerance were set to 20 ppm and 0.05 Da, respectively. Dynamic modifications included Oxidation (+15.995 Da, M), Acetylation (+42.011 Da, N-Term), and static modification included Carbamidomethyl (+57.021 Da, C) and TMT (+229.163 Da, K, N-Term). Peptide-spectrum matches (PSMs) were validated with Percolator, where only PSMs with false discovery rate (FDR)< 0.01 were retained in the analysis.

PSMs were filtered by removing PSMs with average signal-to-noise (SN) ratio lower than 10 and isolation interference higher than 50% and SN was summarized to the protein level for analysis. Protein level data was log_2_-transformed and median normalized, where normalization was performed by taking the median total signal, calculating respective normalization factors for samples and media controls, and then missing values were imputed. Samples were compared against the appropriate media control (HBEC, KSFM; other cell lines, RPMI) on the log_2_ scale, and enriched proteins were determined by analyzing the intersection between sample and media. Differences between technical replicates and cell lines were assessed with principal component analysis (PCA). Proteins were filtered prior to statistical analysis, where only proteins seen in 2 or more technical replicates were retained. The average log_2_ HBEC GFP;p53^wt^ signal intensity was subtracted from all samples to generate average log_2_ fold changes. Sample log_2_ fold changes were analyzed with the limma package (40) (version 3.50.0) with the moderated *t*-test in R, and adjusted *P*-values were calculated with the Benjamini-Hochberg procedure, with those<0.05 considered significant (R version 4.0.5).

### Protein annotation and gene ontology enrichment analysis

The high-throughput model of DeepLoc 2.0 was used for the prediction of subcellular localization for the identified proteins (41). Functioning as a multi-label predictor, it possesses the capability to anticipate one or more localizations for a given protein. Selected gene lists were analyzed for Gene Ontology (GO) terms using the clusterProfiler package in R for enrichment in biological processes, molecular function, and cellular compartments (42). *P-*values were adjusted using the Benjamini-Hochberg procedure and terms with *adjp<* 0.05 were retained (R version 4.0.5). Reactome enrichment analysis was performed using ShinyGO 0.77 with EnsemblIDs corresponding to the individual proteins where available (43, 44). The ‘Curated.Reactome’ database was assessed using default settings, consisting of FDR< 0.05, min pathway size n=2, and max pathway size n=2000 and the top 20 pathways were plotted, sorted by fold enrichment.

### Microarray analysis

Z-score normalized Affymetrix gene expression data collected from 199 primary lung tumors was retrieved from Memorial Sloan Kettering Cancer Center (45). Statistical analysis was performed with the Wilcoxon rank-sum test (Mann-Whitney *U* test) and adjusted *P-*values were calculated using the Benjamini-Hochberg procedure. Analysis was performed using base R functions (version 4.0.5). Statistically significant microarray probes were mapped to their corresponding gene with the R package hgu133a.db (46) (version 3.2.3). Genes were filtered for optimal 1:1 probe:gene mapping with the R package jetset (47) (version 3.4.0) to yield a final gene list.

### Survival analysis

Differentially expressed genes were analyzed for differences in patient survival with NCBI GEO gene expression dataset GSE31210 (48). Probes were mapped to the corresponding genes with the R package hgu133plus2.db (49) (version 3.2.3). Median overall survival was calculated by applying a median split in gene expression and the Logrank test in Graphpad Prism 6.

### ELISA

Quantification of GDF-15 in the secretome was performed using the Human GDF-15 Quantikine ELISA Kit (R&D Systems, DGD150) according to the kit instructions. MDK and SPINT2 were quantified using the Human MDK ELISA Kit (Invitrogen, EH319RB) and SPINT2 (HAI-2) Human ELISA Kit (Invitrogen, EH319RB and EHSPINT2) according to kit instructions. Quantification was performed on conditioned media samples collected under secretome collection conditions, in parallel with secretome experiment collection. All samples were run in duplicate. Sample concentrations were determined by subtracting the media background control signal, then interpolating with a standard curve. Differences in concentrations were statistically computed with the unpaired Student’s T-test in Graphpad Prism6.

### Trypan Blue viability stain

Cells were seeded at approximately 80% confluency in 6-well plates overnight: 350 000 (HBEC); 340,000 (PC-9); 340,000 (H1975); 1,200,000 (HCC4006); 2,400,000 (HCC4011); 850,000 (H3255). Plates were rinsed twice with PBS and media was changed to supplement-free or serum-free conditions and incubated for 24 hours. HBECs were incubated with either supplement-free KSFM and 1% Penicillin-Streptomycin, or KSFM supplemented with BPE (50μg/mL), EGF (5ng/mL), and 1% Penicillin-Streptomycin. NSCLC cell lines were incubated with either serum-free RPMI-1640 and 1% Penicillin-Streptomycin, or RPMI-1640 supplemented with 10% FBS and 1% Penicillin-Streptomycin. Post-incubation, cells were trypsinized with 0.05% Trypsin-EDTA (Thermo Fisher Scientific; HBEC) or 0.25% Trypsin-EDTA (Thermo Fisher Scientific; NSCLC). Trypsinization was neutralized with either trypsin neutralizer (Thermo Fisher Scientific; HBEC) or RPMI-1640 containing 10% FBS (NSCLC), cells mixed in 0.4% Trypan Blue solution (Thermo Fisher Scientific) at a 1:1 ratio, and live cell population determined with a TC20 automated cell counter (Bio-Rad). The average percent live cell population was determined from the average of 3 wells with the unpaired Student’s T-test in Graphpad Prism6 (2 counts per well).

#### Propidium Iodide viability stain

Cells were seeded and treated under supplement-free or serum-free conditions for 24 hours as described in the Trypan Blue viability analysis section. Post-treatment, cells were incubated with 1μg/mL Hoescht 33342 (Thermo Fisher Scientific) for 30 minutes, and 1μg/mL propidium iodide (Thermo Fisher Scientific) for 10 minutes, respectively. Stained cells were imaged with an EVOS FL fluorescence microscope (Thermo Fisher Scientific). Live cell population was determined by quantifying the average live cell population from 2 images per well using ImageJ software, and applying the unpaired Student’s T-test in Graphpad Prism6. The formula 100% − ((PI stained cell population ÷ Hoescht 3342 cell population)×100) was used to determine the live population.

## Results

### Mass-spectrometry secretome profiling of mutant EGFR lung cell models

**Figure 1:**
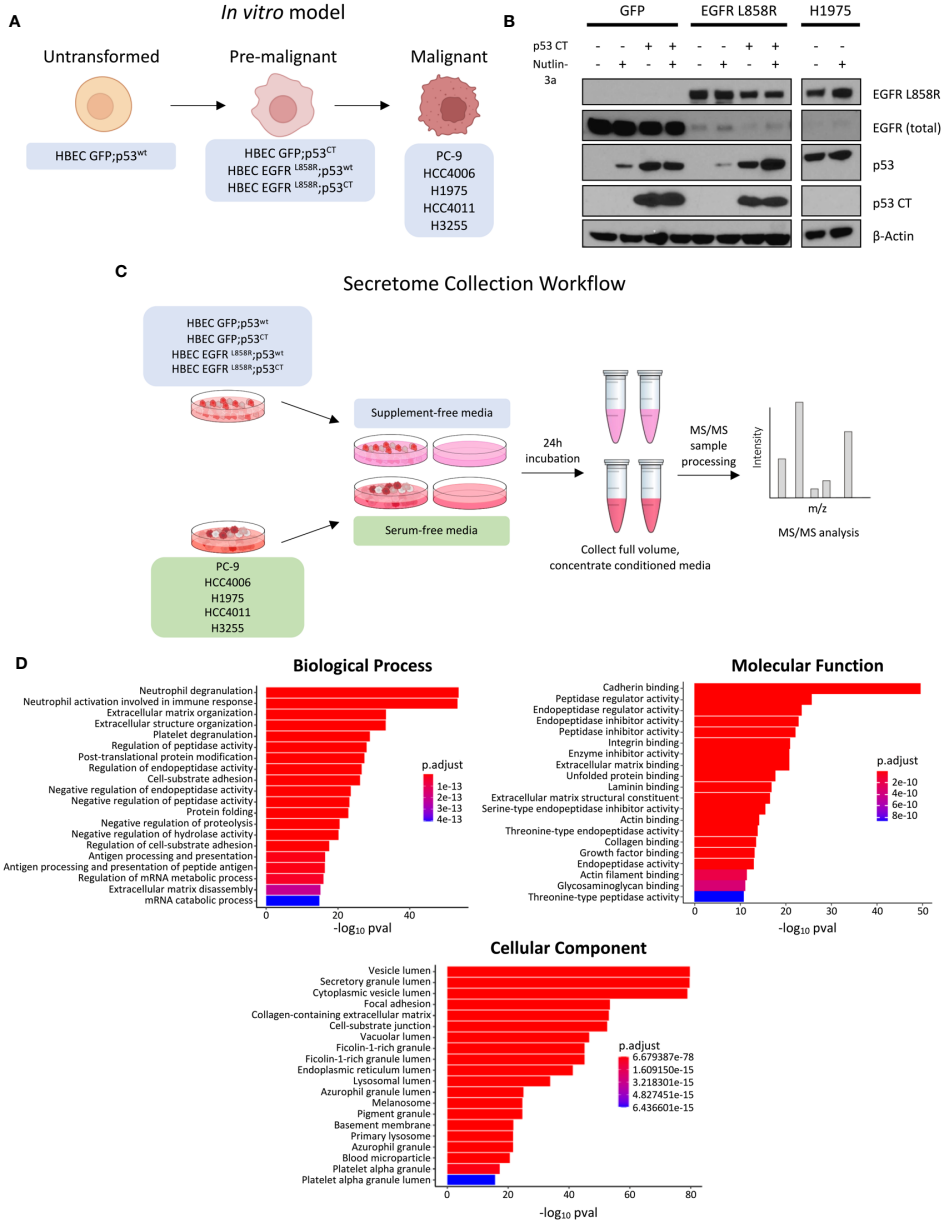
Experiment overview, model expression validation, and GO enrichment analysis of identified proteins. **(A)** Schematic overview of model, where each cell line represents a stage of malignant transformation. HBEC GFP;p53wt represents the wild-type, untransformed state, HBEC cell lines expressing mutations in EGFR-driven transformation represent an intermediate pre-malignant stage, and EGFR mutant NSCLC cell lines PC-9, HCC4006, H1975, HCC4011, and H3255 represent the transformed, malignant state. **(B)** Western blot expression validation of HBEC stable cell lines expressing GFP, EGFR L858R, in combination with p53 CT. Cell lines were treated with 10µM Nutlin 3-a, an MDM2 inhibitor, for 24 hours to confirm mutant p53 expression. EGFR L858R basal expression was also confirmed. H1975 was used as a positive control. **(C)** Schematic overview of the secretome collection experiment. **(D)** Bar plots showing the top 20 GO enrichment terms sorted by adjusted p-value (p adj< 0.05, Benjamini-Hochberg adjustment) for biological processes, molecular functions, and cellular components, for proteins identified in 2 or more technical replicates (prior to statistical analysis).

To study potential changes in secreted proteins during malignant mutant *EGFR*-driven LUAD transformation, we generated an *in vitro* model approximating mutant *EGFR* malignant transformation with HBEC stable cell lines and selected *EGFR* mutant NSCLC cell lines (**Figure 1A**). HBECs, a bronchial epithelial cell line immortalized with non-viral proteins hTERT and CDK4, was selected due to its ability to maintain a non-transformed phenotype post-immortalization *in vitro* and *in vivo* (38). HBEC cell lines stably expressing EGFR^L858R^ or GFP control, with or without dominant negative p53 C-terminal domain alterations (p53^CT^) (GFP;p53^wt,^ GFP;p53^CT^), EGFR^L858R^;p53^wt^, EGFR^L858R^;p53^CT^ represent a profile of commonly mutated genes observed in *EGFR* mutant LUAD (4, 50). To confirm gene expression, HBEC cells were treated with the MDM2 inhibitor Nutlin-3a for 24 hours to assess p53 levels (51). HBEC GFP;p53^CT^ showed minor changes in p53 expression, consistent with a mutant p53 phenotype, comparable to the mutant p53 NSCLC cell line H1975 (52) (**Figure 1B**). Cell lines expressing p53^wt^ showed increased p53 expression, which is consistent with Nutlin treatment (53). Expression of EGFR^L858R^ was confirmed using mutant specific antibodies (**Figure 1B**).

For secretome collection and analysis, HBEC and *EGFR* mutant NSCLC cell lines were starved under supplement-free and serum-free conditions for 24 hours, with cell-free media serving as controls (**Figure 1C**). This was performed to improve the detection of low abundance proteins masked by FBS and minimize non-human contamination, which is common practice in secretome experiments (54). However, starvation conditions can negatively affect cell viability and increase cell cytolysis, potentially contaminating media with intracellular proteins (35, 54). To investigate whether supplement- or serum-free conditions would have an effect in this regard, we assessed cell viability prior to secretome collection (**Supplementary Figures 1**, **2**). Cell lines treated with Alamar Blue or propidium iodide (PI) showed no major changes in cell viability, measured as a live cell population percentage (**Supplementary Figures 2B, C**).

Upon secretome collection, samples were analyzed by TMT-11 MS/MS. To improve detection of low abundance proteins, conditioned media was concentrated prior to mass spectrometry (**Figure 1C**). Data was checked for quality and potential sources of variation introduced during sample processing using principle component analysis (PCA), confirming that technical replicates were generally clustered per cell line tumor or tissue origin, separate from media control samples (**Supplementary Figure 3**). We identified 1020 proteins post-secretome collection. The initial output was filtered to identify proteins in conditioned media by removing the intersection between media control and cell line samples, yielding 852 candidate secreted proteins (see methods). We then assessed this subset for those predicted to contain a signal peptide for secretion and their predicted subcellular localization using DeepLoc 2.0 (41, see methods). This predictive model can distinguish among 10 distinct localization and has the ability to forecast the presence of sorting signal peptides that influence the prediction of subcellular localization.

In total, image 359 (42%) of the candidate proteins are predicted to contain a signal peptide and 217 (26%) are predicted to have extracellular localization (**Supplementary Table 1**). This includes the proteins HSPG2, LAMA5, and AGRN that have previously been demonstrated to be secreted (33). An additional 123 (14%) of the candidate secreted proteins were predicted to be localized to the cell membrane, including EGFR which is known to undergo shedding into the extracellular space (**Supplementary Table 1**) (55).

The enriched protein subset was examined for associated GO terms to further assess if proteins were secreted (**Figure 1D**). Cellular component GO terms were commonly associated with vesicular protein transport; the top enriched terms included “vesicle lumen”, “secretory granule lumen”, and “cytoplasmic vesicle lumen” (**Figure 1D**) (56). Other terms were associated with cellular compartments such as the lysosome and endoplasmic reticulum, which could suggest affiliation with either conventional or unconventional secretion (57, 58). Secretion-associated cellular component terms were complemented by biological process terms that are associated with extracellular proteins, with examples including “neutrophil degranulation”, “extracellular matrix organization”, and “platelet degranulation” (59–61). Of the molecular function GO terms identified, cadherin binding was the most significantly enriched; this may be attributed to cadherin and the associated catenin binding to facilitate cell adhesion (62). Proteolysis terms, such as peptidase and endopeptidase regulation, were also enriched (**Figure 1D**). These terms are consistent with protease functions, which range from cell proliferation to the immune response (63). To interrogate specific signaling pathways associated with the identified proteins we performed a separate enrichment analysis interrogating the Reactome database (see methods). This revealed the top enriched pathways to include post−translational protein phosphorylation, IGF signaling among others including platelet/neutrophil degranulation and non−integrin membrane−ECM interactions that closely resemble the results from the GO analyses (**Supplementary Figure 4**).

### Identification of differentially expressed secreted proteins corresponding to stages of mutant EGFR mediated lung cell transformation

**Figure 2:**
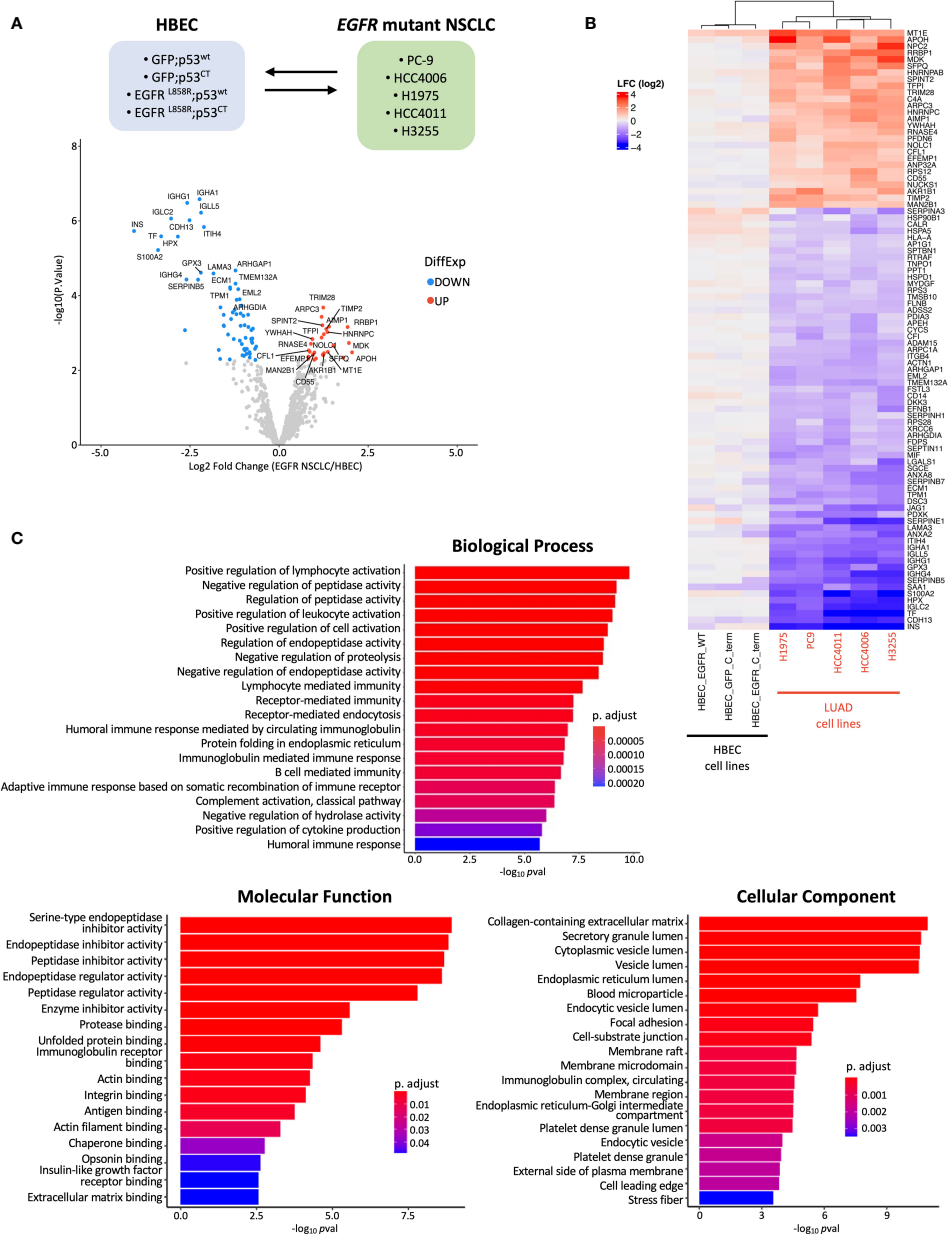
Groupwise comparison between HBEC cell lines and EGFR mutant NSCLC cell lines. **(A)** Schematic showing comparison performed during statistical analysis and volcano plot of log_2_fold change (LFC) differentially expressed proteins identified from MS/MS analysis. The top 20 significantly over- and under-expressed proteins (*p* adj< 0.05 and absolute LFC > 0.6) are colored in red or blue, respectively, and labeled. **(B)** Heatmap of differentially expressed proteins identified from group-wise comparison in A., with hierarchical clustering (n=91) (cell line and protein, hierarchical clustering; cell line clustering distance, complete; protein clustering distance, average). **(C)** Bar plots showing the top 20 GO enrichment terms sorted by adjusted p-value (*p* adj< 0.05, Benjamini-Hochberg adjustment) for biological processes, molecular functions, and cellular components for differentially expressed proteins found during MS/MS analysis.

Differential protein expression (DPE) analysis was performed to investigate differences in secreted proteins between the pre-malignant and malignant stages of *EGFR* mutant LUAD transformation (**Figure 1A**). This was performed by comparing HBEC cell lines expressing mutant *EGFR* and/or p53 to *EGFR* mutant LUAD cell lines and assessing differences specific to each group (**Figure 2A**). 91 proteins were found to be differentially expressed, with 64 under-expressed and 27 over-expressed in the malignant vs non-malignant states, respectively (**Figure 2A**). Hierarchical clustering based on the differentially expressed proteins revealed distinct grouping between HBEC cell lines and *EGFR* mutant NSCLC cell lines (**Figure 2B**). This suggests that there may be distinct secretome profiles between pre-malignant and malignant stages of lung transformation. We assessed the top five over-expressed and under-expressed proteins in malignant vs non-malignant states for their potential as biomarker candidates and found that two, NPC2 and MDK - both of which are predicted to have a signal peptide and extracellular localization (**Supplementary Table 1**) – have been found to be over-expressed in mouse LUAD plasma and NSCLC patient serum, respectively, confirming their secretion (64, 65). We queried the 91 differentially expressed proteins for GO terms associated with biological processes, molecular functions, and cellular components (**Figure 2C**). Four of the top five enriched cellular compartment GO terms were associated with secretory pathways, such as “secretory granule lumen” and “cytoplasmic vesicle lumen”, suggesting the presence of secreted proteins or proteins involved in secretion; this includes conventional secretion, but also unconventional secretion mediated by lysosomes, autophagosomes, and multivesicular bodies that become exosomes (58, 66). “Collagen-containing extracellular matrix” was the top enriched cellular component GO term, which could reflect the predominant role of collagen in extracellular matrix formation and integrity, as well as functions such as cell adhesion (67, 68). Similar to the observations made during initial secretome profiling, terms related to proteolysis represented the top 5 molecular function terms (**Figures 1B**, **2C**). This is reflective of the broad functions of proteases which include extracellular matrix assembly and remodeling and aligns with the top GO cellular component terms (69). Likewise, protease-related functions were represented in biological process GO terms; interestingly, immune cell functions were also represented (**Figure 1B**). The presence of immune cell-related terms suggests changes in immune regulatory programs that occur during transformation. This aligns with previous observations where PD-L1, a key protein in immune homeostasis, was upregulated in *EGFR* mutant NSCLC cell lines and expression associated with clinical LUAD samples (70, 71). Reactome analysis also revealed enrichment in numerous immune related signaling pathways in addition to ATF6 and JAK-STAT signaling (**Supplementary Figure 5**).

To investigate the differences in secreted proteins during transformation, we analyzed differences in secreted proteins between untransformed and pre-malignant stages (**Figure 1A**, **Supplementary Figure 6**). This was done through comparison across the HBEC cell lines expressing different mutant proteins. DPE analysis was performed where HBEC GFP;p53^CT^, HBEC EGFR^L858R^;p53^wt^, and HBEC EGFR^L858R^;p53^CT^ were individually compared to HBEC GFP;p53^wt^ cells. In contrast to the malignant vs non-malignant comparison, only PFKP, FN1, SERPINA3, and SERPINB7 were found to be differentially expressed in the pre-malignant and non-transformed states (**Supplementary Figures 6A–C**). SERPINA3 and SERPINB7 were also differentially expressed in the malignant vs non-malignant comparison (**Figure 2B**). This suggests that their expression levels may change during multiple stages of transformation. As few differentially secreted proteins were identified through this analysis, it is possible that expression of cancer genes alone is insufficient to dramatically alter the secretome or that the pre-malignant stage of transformation may not be distinct from the histologically normal, untransformed stage in terms of secreted profiles.

### Identification of secreted biomarker candidates specific to the malignant state

**Figure 3:**
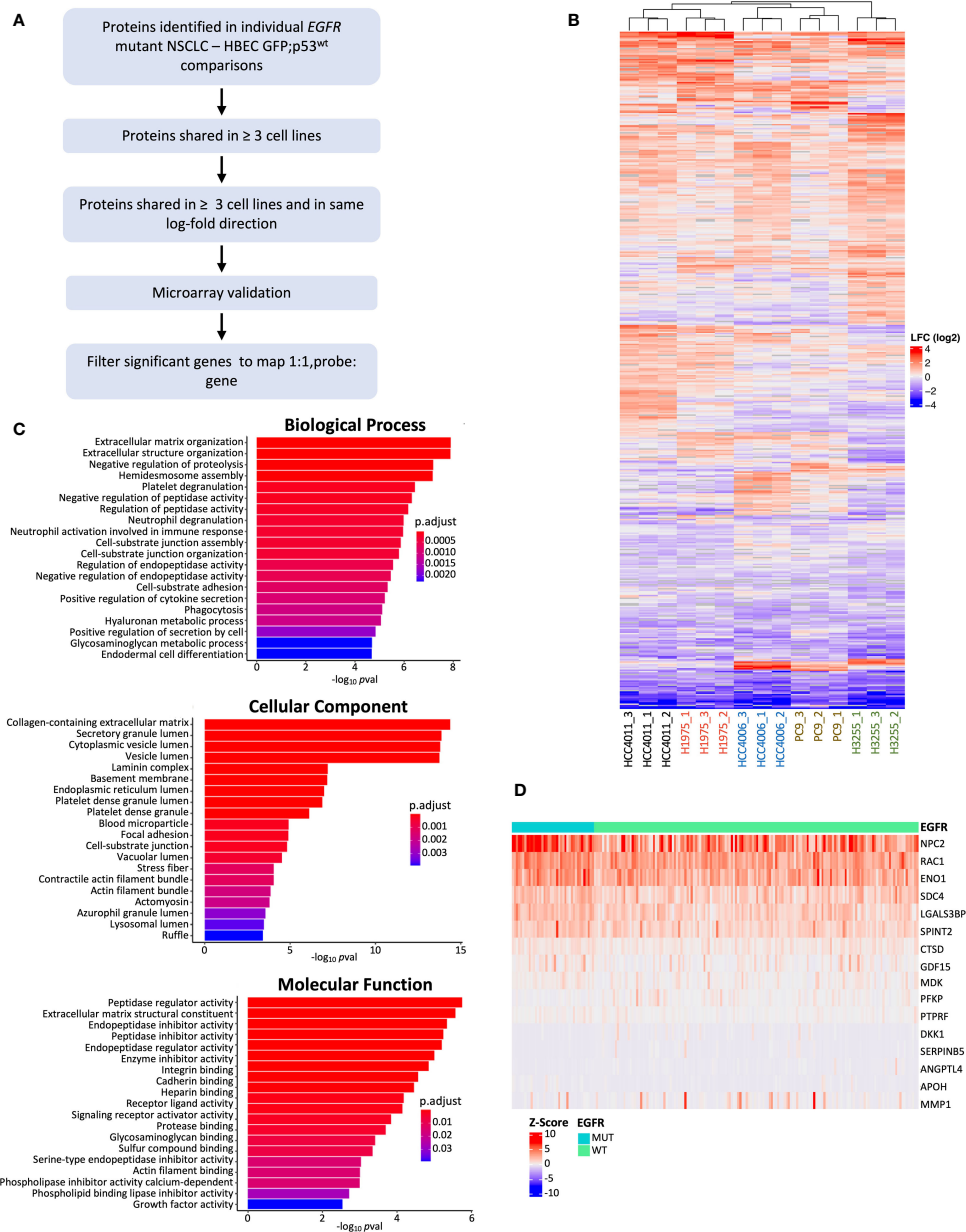
Individual EGFR mutant NSCLC cell line comparison against HBEC GFP;p53^wt^ and filtering pipeline. **(A)** Filtering pipeline used to identify protein candidates for biomarker validation. Differentially expressed proteins from cell line comparisons with HBEC GFP;p53^wt^ (*p* adj< 0.05, absolute LFC > 0.6) were determined, and filtered for microarray gene expression validation if found in 3 or more *EGFR* mutant cell line comparisons and LFC expression was in the same direction (LFC values were all positive or negative). Filtered differentially expressed proteins were analyzed for differential gene expression with Z-score normalized Affymetrix gene expression data from 199 primary LUAD tumors (45), and differentially expressed genes that were found to be significant (*p* adj*<* 0.05, Benjamini-Hochberg adjustment) were further filtered for optimal 1:1 probe to gene mapping for additional stringency (47). **(B)** Heatmap of differentially expressed genes found from individual comparison between *EGFR* mutant NSCLC cell lines and HBEC GFP;p53^wt^ (cell line and protein, hierarchical clustering; cell line clustering distance, complete; protein clustering distance, average). LFC ranges from high (red) to low (blue). **(C)** Bar plots showing the top 20 GO enrichment terms sorted by adjusted p-value (*p* adj< 0.05, Benjamini-Hochberg adjustment) for biological processes, molecular functions, and cellular components for differentially expressed proteins found during individual cell line comparisons between *EGFR* mutant NSCLC cell lines and HBEC GFP;p53^wt^. **(D)** Heatmap of differentially expressed genes post-microarray analysis for further validation (*p* adj< 0.05). Samples are grouped by EGFR status (mutant, wild type) (gene clustering method, Euclidean; gene clustering distance, complete).

To identify protein candidates for further analysis and validation, we also performed DPE analysis of *EGFR* mutant NSCLC cell lines individually against HBEC GFP;p53^wt^, defined as the initial, untransformed stage (**Figure 1A**). This was done to capture cell line-specific differentially expressed proteins not observed in a group-wise comparison. **Figure 3A** outlines the filtering pipeline to identify potential protein biomarker candidates using p adj< 0.05 and LFC > 0.6. The number of differentially expressed proteins ranged from 119 to 316 per LUAD cell line, in contrast to 91 from the group-wise comparison (**Supplementary Figure 7**). Individual comparisons may highlight specific genetic alterations per cell line, as each cell line has varying *EGFR* and *TP53* mutations (72–74). Clustering based on differentially expressed proteins demonstrated that EGFR L858R driver mutation cell lines HCC4011 and H1975 grouped together, while EGFR exon19 deletion cell lines HCC4006 and PC-9 clustered together. The exception was H3255, an EGFR L858R mutant, which may be attributed to other differences in genetic alterations; an example is H1975 possessing CDKN2A and PIK3CA mutations that are not found in H3255 (74, 75). We then profiled the 499 differentially expressed proteins found across all LUAD cells compared to the non-transformed state for enrichment in GO terms associated with biological processes, molecular functions, and cellular compartments (**Figure 3C**). Similar to the group-wise comparison, GO cellular component terms were associated with the extracellular matrix, such as “collagen-containing extracellular matrix”, “laminin complex”, and “basement membrane” (60, 76). Also common were secretion associated terms, with “secretory granule lumen”, “cytoplasmic lumen”, and “vesicle lumen” comprising 3 of the top 5 cellular component terms (**Figure 3D**) (58, 66). Reactome analysis revealed enrichment in similar signaling pathways as the groupwise comparison, with the noted addition of MET related signaling as one of the most enriched pathways (**Supplementary Figure 8**).

Proteins identified from the individual cell line comparisons were filtered for further analysis (**Figure 3A**). Differentially expressed proteins were filtered for overlap in three or more *EGFR* mutant LUAD cell lines, and then for expression in the same direction. This resulted in 130 proteins for further investigation. As there was no relevant *EGFR* mutant LUAD proteomic dataset available, we aimed to assess whether the expression of the secreted proteins are specific to EGFR mutant LUADs using transcriptomic data. Differential gene expression analysis was performed on a cohort of 39 *EGFR* mutant and 154 *EGFR* wild-type tumors (45). This analysis revealed 16 genes differentially expressed between EGFR mutant and wild-type LUAD tumors with corresponding proteins that were differentially secreted in the EGFR mutant LUAD cell lines (**Table 1**, **Figure 3D**). NPC2 demonstrated the highest level of expression, consistent with previous findings where NPC2 expression was greater in LUAD compared to other lung tumor types (77, 78). ENO1 and RAC1 also showed high levels of expression in EGFR mutant LUADs (**Figure 3D**) aligning with previous studies demonstrating that ENO1 expression is greater in LUAD tumor samples relative to non-cancerous tissue, and the RAC1 splice variant RAC1B enhances LUAD tumor formation *in vivo* (79, 80). The analysis of our MS/MS results identified differentially expressed proteins from our secretome experiment with evidence that they may be useful candidate biomarkers in the clinical setting.

**Table 1:** Protein candidates identified from microarray gene expression analysis, listed by gene symbol, sorted by adjusted p value (p adj< 0.05; Benjamini-Hochberg adjustment).

Accession	Gene	Peptides	Unique peptides	Quantified peptides	adj.P.Val
Q08380	LGALS3BP	23	23	20	9.5519E-05
P61916	NPC2	10	10	10	9.5519E-05
O43291	SPINT2	2	2	1	0.001714715
P06733	ENO1	30	28	27	0.003759408
P21741	MDK	12	12	11	0.003759408
P63000	RAC1	3	2	1	0.005590461
Q01813	PFKP	2	2	1	0.007683377
P31431	SDC4	7	7	5	0.012165019
Q99988	GDF15	6	6	4	0.020488018
P02749	APOH	2	2	2	0.02368962
P36952	SERPINB5	14	14	8	0.027201045
P10586	PTPRF	19	19	15	0.031704624
P07339	CTSD	20	20	20	0.034872449
O94907	DKK1	7	7	7	0.034872449
P03956	MMP1	2	2	2	0.035149978
Q9BY76	ANGPTL4	6	6	4	0.03699355

### Secreted proteins with gene expression levels associated with poor outcome in EGFR mutant LUAD

**Figure 4:**
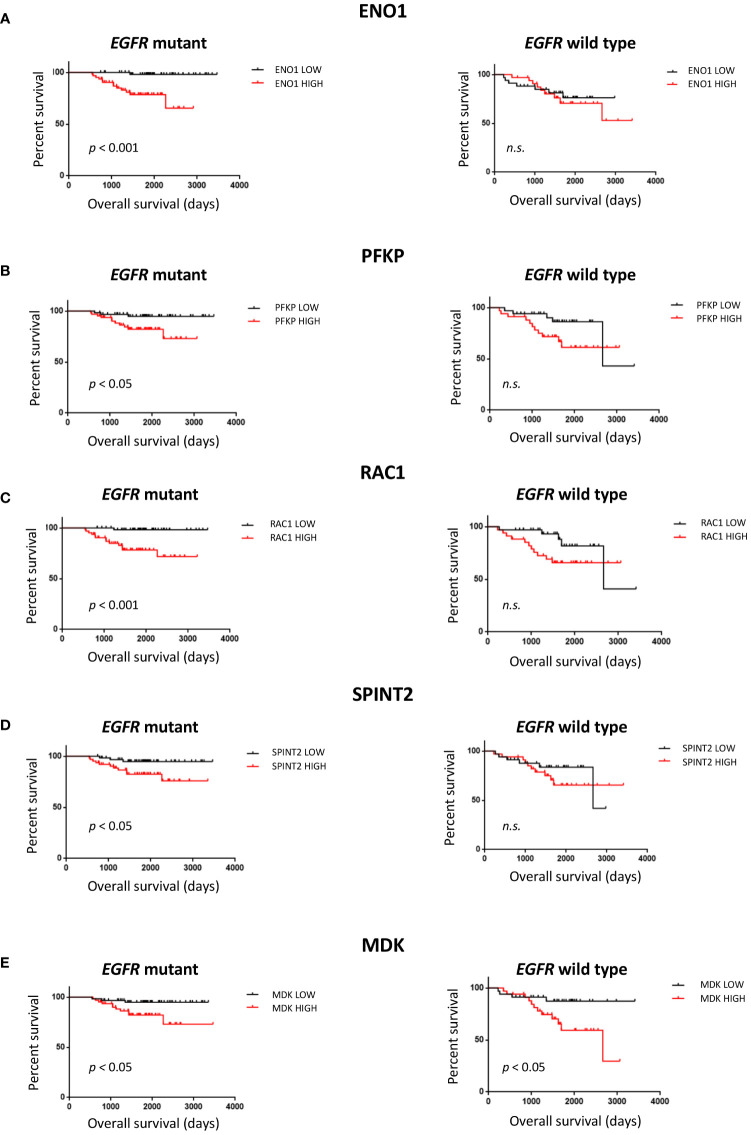
Survival analysis of protein candidates identified from filtering pipeline. Overall patient survival was analyzed (log rank test, median split) for *EGFR* mutant tumors, n=125 and *EGFR* wild type tumors, n=68 from the NCBI GEO GSE 31219 dataset. **(A)** ENO1. **(B)** PFKP **(C)** RAC1 **(D)** SPINT2 **(E)** MDK. *n.s.* non-significant.

The 16 protein candidates found to have EGFR specific expression levels in LUAD tumors were subsequently analyzed for survival difference between 125 *EGFR* mutant and 68 wild-type patient tumors in an independent dataset (**Table 1**) (48). Kaplan-Meier curves were plotted, where overall survival duration between patients with high and low expression of genes was compared based on *EGFR* mutation status (**Figure 4**). High expression of *ENO1 (p<* 0.001), *PFKP* (*p<* 0.05), *RAC1 (p<* 0.001), and *SPINT2* (*p<* 0.05) in patients with *EGFR* mutant tumors was associated with shorter overall survival than *EGFR* mutant tumors with low gene expression (**Figures 4A–D**). The association with poor survival was not seen in patients with *EGFR* wild-type tumors, suggesting that survival differences could be *EGFR* mutation-specific. High *MDK* expression was also associated with lower overall survival, in *EGFR* mutant and wild-type patient tumors (*p<* 0.05) (**Figure 4E**). This observation aligns with a previous study where NSCLC patients displayed increased protein expression of serum MDK compared to healthy individual controls, and expression was associated with lower overall survival (65).

### Orthogonal validation of secreted proteins

**Figure 5:**
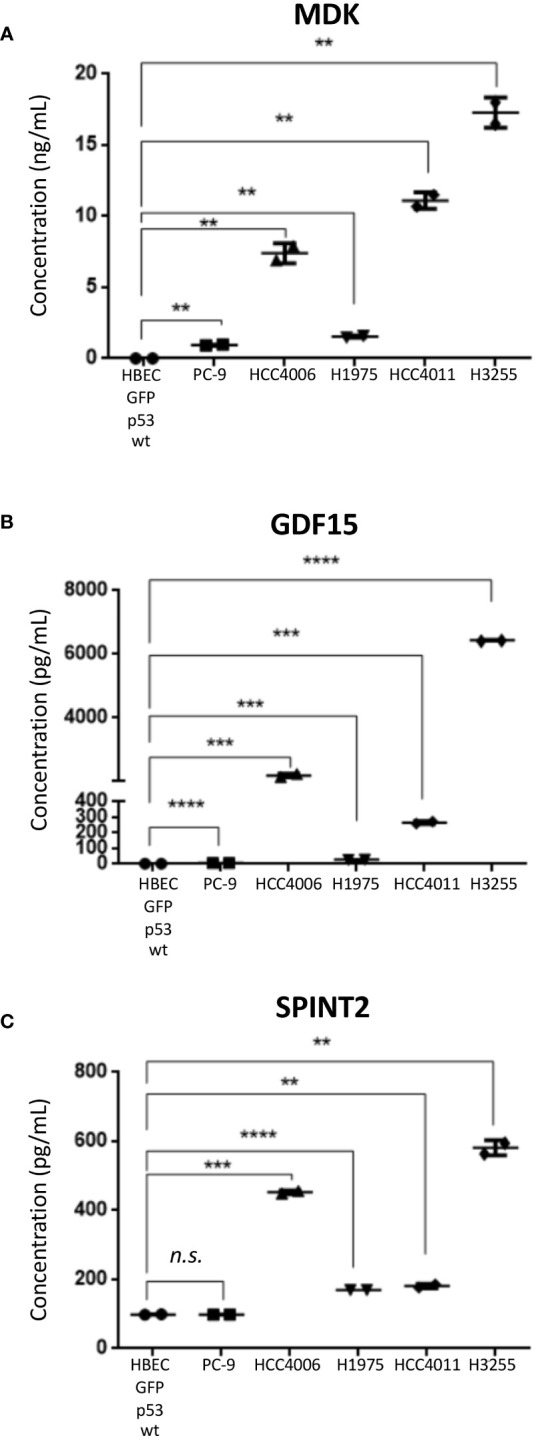
ELISA analysis of selected protein candidates in secretome conditioned media. Mean values ± SD are shown, experiment performed in technical duplicate (Student’s unpaired T-test, two tailed). **(A)** MDK **(B)** GDF15 **(C)** SPINT2. ** *p*< 0.01, *** *p*< 0.001, **** *p*< 0.0001, *n.s.* non-significant.

To validate levels of secreted protein expression in the EGFR mutant LUAD cell lines, MDK, GDF15, and SPINT2 were assessed by ELISA assays (**Table 1**, **Figure 5**). MDK and SPINT2 were selected due to the observed differences in overall survival while GDF15 levels have been associated with different stages of lung cancer in patients (81). Protein concentration was measured in conditioned media samples collected and concentrated in parallel with the MS/MS samples. As we were interested in comparing between malignant and untransformed, histologically normal states, we compared protein concentration between HBEC GFP;p53^wt^ and the selected LUAD *EGFR* mutant cell lines (**Figure 5**). The mean MDK concentration in LUAD cell lines ranged from 0.94 - 17.28 ng/mL, and when compared to HBEC GFP;p53^wt^, concentrations were significantly different (*p<* 0.01; **Figure 5A**). GDF15 mean protein concentration in NSCLC cell lines varied between 5.85 pg/mL - 6.44 ng/mL, compared to the mean HBEC GFP;p53^wt^ concentration of 2.41 pg/mL. This corresponded to an increase of secreted GDF15 concentration in NSCLC cell lines up to 2000x that observed in HBEC GFP;p53^wt^ (PC-9, *p<* 0.0001; HCC4006, *p*< 0.001; H1975, *p<* 0.001; HCC4011, *p*< 0.001; H3255, *p<* 0.0001; **Figure 5B**). SPINT2 mean protein concentration in NSCLC cell lines ranged between 98.30 - 580.97 pg/mL, relative to 98.58 pg/mL in HBEC GFP;p53^wt^. With the exception of PC-9, protein concentrations were significantly greater than HBEC GFP;53^wt^, where concentrations were 1.7- 5.9 times greater (HCC4006, *p*< 0.001; H1975, *p<* 0.0001; HCC4011, *p*< 0.01; H3255, *p<* 0.01; **Figure 5C**). Together, these assays confirm the MS results, validating the increased secretion in EGFR mutant LUAD.

## Discussion

Earlier detection of LUAD is key to long-term patient survival, where LDCT screening could benefit from the inclusion of biomarkers to complement screening (12). This is especially important given that LDCT screening for never smokers, which have increased incidence of *EGFR* mutant LUAD, does not have concrete guidelines (20, 37). Compared to ever smokers, one study found that the rate of diagnosis with LDCT screening for a never smoker cohort was 0.45%, which was lower than the NLST ever smoker rate of 1.0% (18, 82). More recently, a LDCT screening study for primarily non-smoking Asian women, the demographic commonly associated with *EGFR* mutant LUAD, found an increase in cancer incidence in early-stage cancers (stages 0-I) yet no change in late-stage incidence (stages II-IV) (83). These findings suggested that additional cases identified by LDCT were attributed to over-diagnosis, and that LDCT would have limited use for populations affected by *EGFR* mutant LUAD, furthering the necessity of biomarker-based detection to inform clinical decisions (21, 83). Currently, NSCLC biomarker candidates are limited and are neither specific for lung cancer nor histology (23). The cancer secretome is a valuable resource to uncover prognostic and diagnostic biomarker candidates (25, 84).

To date, secretome studies focusing on NSCLC have primarily analyzed established cancer cell lines, identifying changes in secreted proteins that provide further insight into processes such as tumor growth and metastasis (33, 85). However, cancer cell lines may not capture changes that occur during earlier stages of malignant transformation, resulting in missing potential biomarkers for earlier detection. Using an *in vitro* model representative of malignant transformation, this study analyzed the secretome of *EGFR*-driven LUAD malignant transformation *in vitro*. Our study began with generating a model and serum-free culture conditions compatible with mass spectrometry analysis to profile the secretome. Due to the lack of a transformation phenotype *in vitro* and *in vivo*, the HBEC cell line was selected to serve as an untransformed basal state to compare secretome changes during different transformation stages (38). By introducing common genetic alterations found in *EGFR* and *p53* into HBECs, we could also profile secretome changes during an intermediate, pre-malignant stage of transformation (50, 86, 87). An additional benefit to the HBEC cell line was its ability to be cultured in non-serum conditions; serum proteins often obscure low abundance proteins and may introduce non-human contamination (33, 54).

Together with selected *EGFR* mutant NSCLC cell lines representing the transformed state, we generated a model that represents key genetic alterations occurring during *EGFR*-driven malignant transformation (**Figures 1A, B**) (9, 50). We initially identified 1020 proteins, where 852 proteins from secretome conditioned media were enriched. HSPG2, LAMA5, and AGRN were identified which is consistent with a previous NSCLC secretome study, suggesting that secreted proteins could be detected with our approach (33). We further validated the presence of secreted proteins by performing GO analyses, where cellular component terms referenced secretory pathways or locations associated with secretion (**Figure 1D**) (58, 66). We identified 91 differentially expressed proteins in *EGFR* mutant NSCLC cell lines relative to HBEC cell lines (**Figures 2A, B**) with secretome profiles of the transformed states including changes in immune functions. This aligns with previous NSCLC studies where EGFR mutations were associated with immune changes such as increased expression of PD-1 and PD-L1, and decreased CD8+ T cell infiltration (**Figure 2C**) (88, 89). We were unable to identify notable differences when comparing the basal HBEC GFP;p53^wt^ cell line to HBEC cell lines expressing *EGFR* and *p53* alterations (**Supplementary Figure 5**) suggesting the introduction of additional genetic alterations may be needed to establish a more advanced pre-malignant state (9).

To broaden the pool of potential candidates and identify the most notable differences between untransformed and transformed states, we also compared *EGFR* mutant NSCLC cell lines individually to the basal HBEC cell line (**Figure 3**). 499 proteins across all comparisons were identified, with 130 proteins differentially expressed (**Figures 3A, B**). The secretome profile of the LUAD cell lines had a broad scope of biological functions (**Figure 3C**) which may be due to additional genetic changes specific to each NSCLC cell line, beyond EGFR and p53, such as p16 that can affect the secretome profile (90, 91). Using transcriptome data, we found genes for 16 of these proteins which were expressed specifically in EGFR mutant LUAD, suggesting they may be biomarker candidates for this molecular subtype of lung cancer (**Figure 3D**) (65, 78). A subset of these were also associated with patient survival in EGFR mutant LUAD, and we validated MDK, GDF15, and SPINT2 by ELISA, confirming their secretion and association with the malignant state.

MDK is a growth factor that binds to heparin and is involved in promoting cell growth and survival *in vitro* and tumor growth *in vivo* in a model of LUAD (92). GDF15 is a member of the transforming growth factor-β superfamily and varying biological effects have been observed with expression changes (93). In one study, GDF15 overexpression suppressed cell proliferation *in vitro* and tumor formation *in vivo*, while in another study overexpression promoted tumor growth *in vivo*, and proliferation *in vitro* when stimulated with C5a (94, 95). SPINT2 is a serine protease inhibitor where decreased expression facilitated STYK1-mediated tumor progression (96). With the exception of the PC-9 cell line when measuring SPINT2 concentration, *EGFR* mutant NSCLC cell lines had significantly higher concentrations of the selected proteins than HBEC GFP;p53^wt^ (**Figure 5**). This suggests that there may be changes in MDK, GDF15, and SPINT2 expression during *EGFR*-driven malignant transformation that could be indicative of progression and studied for biomarker use (81, 97, 98).

While our study sought to identify changes in the secretome during LUAD transformation *in vitro*, there are limitations that should be considered for future studies. Firstly, the HBEC cell lines which represented the pre-malignant model stages were not validated for transformation capacity anchorage-independent growth *in vitro* or growth *in vivo* (99). As a result, this hampered the accuracy of the model when compared to clinical stepwise transformation and thus the accuracy of secretome changes occurring during the pre-malignant stage (50). Secondly, there may be additional genetic alterations that occur during EGFR-driven transformation. A previous study modeling transformation in HBECs found that *EGFR* and *TP53* mutations were unable to promote transformation *in vivo*, while another identified that alterations in APC, RB1, and RBM10 promoted tumor growth *in vivo* (9, 100). Thirdly, limited incubation time under serum-free conditions can restrict the scope of secretome profiling, as secretome protein abundance has been observed to increase over time, despite minimizing cell death (35, 101, 102).

## Conclusions

In summary, we have profiled the secretome of non-transformed and EGFR mutant transformed lung cells and identified 3 protein candidates that were validated for differential expression in EGFR mutant patients. These proteins show promise as candidates for lung cancer biomarker applications, although further mechanistic and validation studies are needed. The data and findings shown provide an insight into secretome changes under a variety of conditions and will serve as a valuable resource to support future studies in LUAD biomarker discovery and molecular changes occurring during EGFR-driven malignant transformation.

## Data availability statement

The original contributions presented in the study are publicly available. This data can be found here: [ProteomeXchange/PXD045328].

## Ethics statement

Ethical approval was not required for the studies on humans in accordance with the local legislation and institutional requirements because only commercially available established cell lines were used.

## Author contributions

JL: Conceptualization, Data curation, Formal analysis, Investigation, Methodology, Validation, Visualization, Writing – original draft, Writing – review & editing. FJ: Investigation, Methodology, Writing – review & editing. JJ: Data curation, Formal analysis, Investigation, Methodology, Validation, Visualization, Writing – review & editing. TS: Investigation, Methodology, Writing – review & editing. RS: Investigation, Methodology, Writing – review & editing. DL: Investigation, Methodology, Writing – review & editing. DF: Investigation, Methodology, Writing – review & editing. SS: Investigation, Methodology, Project administration, Writing – review & editing. GL: Investigation, Methodology, Writing – review & editing. GM: Project administration, Supervision, Writing – review & editing. WL: Conceptualization, Funding acquisition, Project administration, Resources, Supervision, Writing – original draft, Writing – review & editing.

## Supplementary material

Supplementary Figure 1
*Cell viability analysis of HBEC stable cell lines under secretome conditions.* HBEC cell lines were seeded and incubated overnight, then media was changed to secretome media conditions (KSFM, 1% PenStrep) or standard culture conditions (KSFM, supplemented with BPE, EGF, and 1% PenStrep) for 24 hours, then cell viability was assessed with Trypan Blue and Propidium Iodide (PI) staining. **(A)** Quantification of HBEC live cell population, as stained with Trypan Blue. **(B)** Quantification of HBEC live cell population, as stained with PI. **(C)** Representative images of DAPI and PI channels used to quantify PI staining. Experiment was performed in biological triplicate. *n.s.* non-significant.

Supplementary Figure 2
*Cell viability analysis of EGFR mutant NSCLC cell lines under secretome conditions.* Cell lines were seeded and incubated overnight, then media was changed to secretome media conditions (RPMI, 1% PenStrep) or standard culture conditions (RPMI, supplemented with 10% FBS, and 1% PenStrep) for 24 hours, then cell viability was assessed with Trypan Blue and Propidium Iodide (PI) staining. **(A)** Quantification of live cell population, as stained with Trypan Blue. **(B)** Quantification of live cell population, as stained with PI. **(C)** Representative images of DAPI and PI channels used to quantify PI staining. Experiment was performed in biological triplicate. ** *p*< 0.01, *n.s.* non-significant.

Supplementary Figure 3
*Secretome experiment PCA*. PCA was performed on all proteins identified during MS/MS **(A)** PCA excluding media control samples. **(B)** PCA including media control samples.

Supplementary Figure 4 Reactome pathway analysis of candidate secreted proteins from all cell lines. Plot showing the top 20 pathways identified using the Curated.Reactome database (FDR< 0.05). Minimum pathway size was n = 2, maximum pathway size n = 2000.

Supplementary Figure 5 Groupwise Reactome analysis of secreted proteins between HBEC cell lines and EGFR mutant NSCLC cell lines. Plot showing the top 20 pathways identified using the Curated.Reactome database (FDR< 0.05). Minimum pathway size was n = 2, maximum pathway size n = 2000.

Supplementary Figure 6 Differential protein expression analysis of HBEC cell lines expressing EGFR L858R, with or without expression of p53 c-terminal, relative to HBEC GFP;p53^wt^ with different absolute LFC parameters to identify differentially expressed proteins. The top 20 significantly over- and under-expressed proteins (p adj< 0.05 and absolute LFC > 0.6) are colored in red or blue, respectively, and labeled. **(A)** HBEC GFP;p53^CT^ i) minimum absolute LFC< 0.6 ii) minimum absolute LFC< 0.3 **(B)** HBEC EGFR^L858R^;p53^wt^ i) minimum absolute LFC< 0.6 ii) minimum absolute LFC< 0.3 **(C)** HBEC EGFR^L858R^;p53^CT^ i) minimum absolute LFC< 0.6 ii) minimum absolute LFC< 0.3 **(D)** Venn diagram describing overlap in differentially expressed proteins among HBEC stable cell lines. “abs” = absolute.

Supplementary Figure 7
*Differential protein expression analysis of EGFR mutant NSCLC cell lines, relative to HBEC GFP;p53^wt^.* The top 20 significantly over- and under-expressed proteins (*p* adj< 0.05 and absolute LFC > 0.6) are colored in red or blue, respectively, and labeled. **(A)** PC-9. **(B)** HCC4006. **(C)** H1975 **(D)** HCC4011 **(E)** H3255.

Supplementary Figure 8 Reactome pathway enrichment of proteins detected in individual EGFR mutant NSCLC cell lines vs HBEC GFP;p53^wt^. Plot showing the top 20 pathways identified using the Curated.Reactome database (FDR< 0.05). Minimum pathway size was n = 2, maximum pathway size n = 2000.
