# Supplementary material for: Characterizing the secretome of EGFR mutant lung adenocarcinoma
Source: Front Oncol. 2024 Jan 8;13:1286821. doi: 10.3389/fonc.2023.1286821 (PMC10801028; doi:10.3389/fonc.2023.1286821)
Supplement: Supplementary Figure 1 — Cell viability analysis of HBEC stable cell lines under secretome conditions. HBEC cell lines were seeded and incubated overnight, then media was changed to secretome media conditions (KSFM, 1% PenStrep) or standard culture conditions (KSFM, supplemented with BPE, EGF, and 1% PenStrep) for 24 hours, then cell viability was assessed with Trypan Blue and Propidium Iodide (PI) staining. (A) Quantification of HBEC live cell population, as stained with Trypan Blue. (B) Quantification of HBEC live cell population, as stained with PI. (C) Representative images of DAPI and PI channels used to quantify PI staining. Experiment was performed in biological triplicate. n.s. non-significant. [file DataSheet_1.docx]

**Supplementary Figures**

**Figure S1. *Cell viability analysis of HBEC stable cell lines under secretome conditions.*** HBEC cell lines were seeded and incubated overnight, then media was changed to secretome media conditions (KSFM, 1% PenStrep) or standard culture conditions (KSFM, supplemented with BPE, EGF, and 1% PenStrep) for 24 hours, then cell viability was assessed with Trypan Blue and Propidium Iodide (PI) staining. **A.** Quantification of HBEC live cell population, as stained with Trypan Blue. **B.** Quantification of HBEC live cell population, as stained with PI**. C.** Representative images of DAPI and PI channels used to quantify PI staining. Experiment was performed in biological triplicate. *n.s.* non-significant.

**Figure S2. *Cell viability analysis of EGFR mutant NSCLC cell lines under secretome conditions.*** Cell lines were seeded and incubated overnight, then media was changed to secretome media conditions (RPMI, 1% PenStrep) or standard culture conditions (RPMI, supplemented with 10% FBS, and 1% PenStrep) for 24 hours, then cell viability was assessed with Trypan Blue and Propidium Iodide (PI) staining. **A.** Quantification of live cell population, as stained with Trypan Blue. **B.** Quantification of live cell population, as stained with PI. **C.** Representative images of DAPI and PI channels used to quantify PI staining. Experiment was performed in biological triplicate. ** *p* < 0.01, *n.s.* non-significant.

**Figure S3.** ***Secretome experiment PCA***. PCA was performed on all proteins identified during MS/MS **A.** PCA excluding media control samples. **B.** PCA including media control samples.


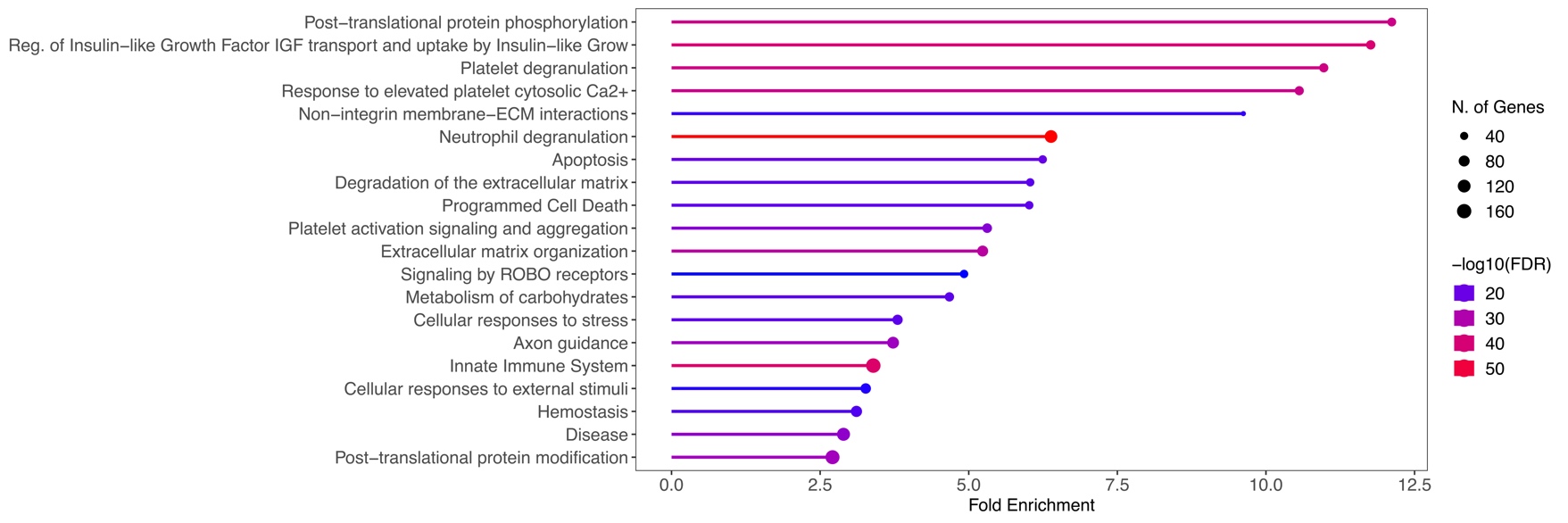


**Figure S4: Reactome pathway analysis of candidate secreted proteins from all cell lines.** Plot showing the top 20 pathways identified using the Curated.Reactome database (FDR < 0.05). Minimum pathway size was n = 2, maximum pathway size n = 2000.


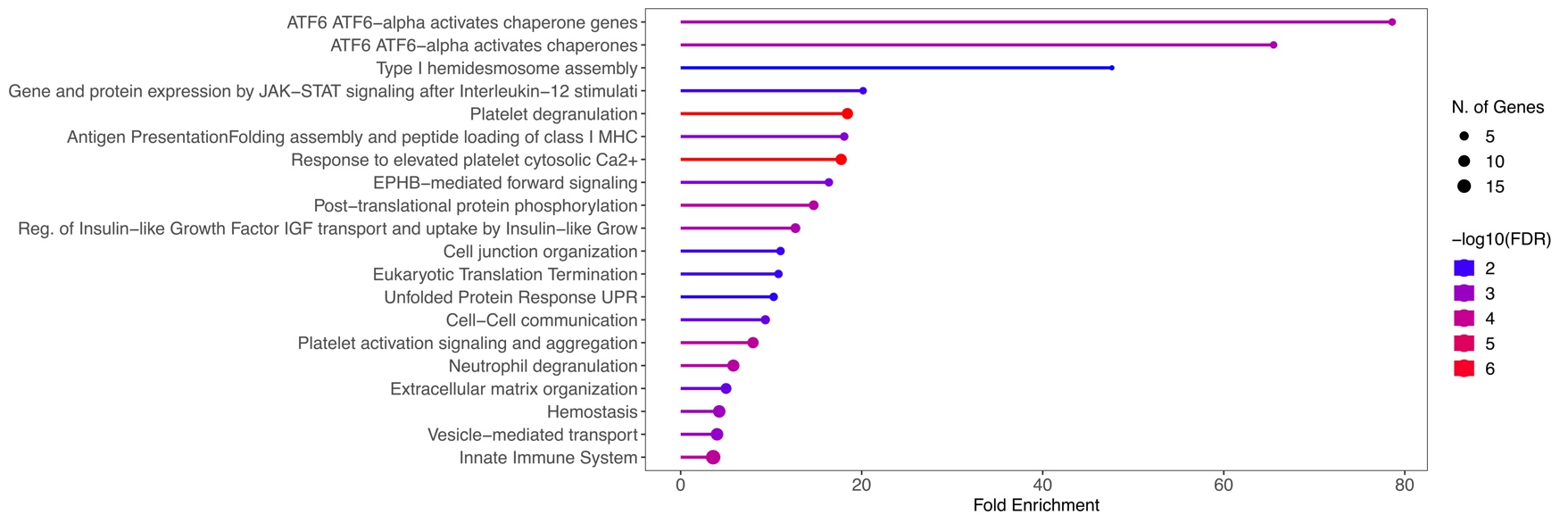


**Figure S5: Groupwise Reactome analysis of secreted proteins between HBEC cell lines and EGFR mutant NSCLC cell lines.** Plot showing the top 20 pathways identified using the Curated.Reactome database (FDR < 0.05). Minimum pathway size was n = 2, maximum pathway size n = 2000.

**Figure S6.** ***Differential protein expression analysis of HBEC cell lines expressing EGFR L858R, with or without expression of p53 c-terminal, relative to HBEC GFP;p53^wt^ with different absolute LFC parameters to identify differentially expressed proteins.*** The top 20 significantly over- and under-expressed proteins (*p* adj < 0.05 and absolute LFC > 0.6) are coloured in red or blue, respectively, and labeled. **A.** HBEC GFP;p53^CT^ **i)** minimum absolute LFC < 0.6 **ii)** minimum absolute LFC < 0.3 **B.** HBEC EGFR^L858R^;p53^wt^ **i)** minimum absolute LFC < 0.6 **ii)** minimum absolute LFC < 0.3 **C.** HBEC EGFR^L858R^;p53^CT^ **i)** minimum absolute LFC < 0.6 **ii)** minimum absolute LFC < 0.3 **D.** Venn diagram describing overlap in differentially expressed proteins among HBEC stable cell lines. “abs” = absolute.

**Figure S7. *Differential protein expression analysis of EGFR mutant NSCLC cell lines, relative to HBEC GFP;p53^wt^.*** The top 20 significantly over- and under-expressed proteins (*p* adj < 0.05 and absolute LFC > 0.6) are coloured in red or blue, respectively, and labeled. **A**. PC-9. **B**. HCC4006. **C.** H1975 **D.** HCC4011 **E.** H3255


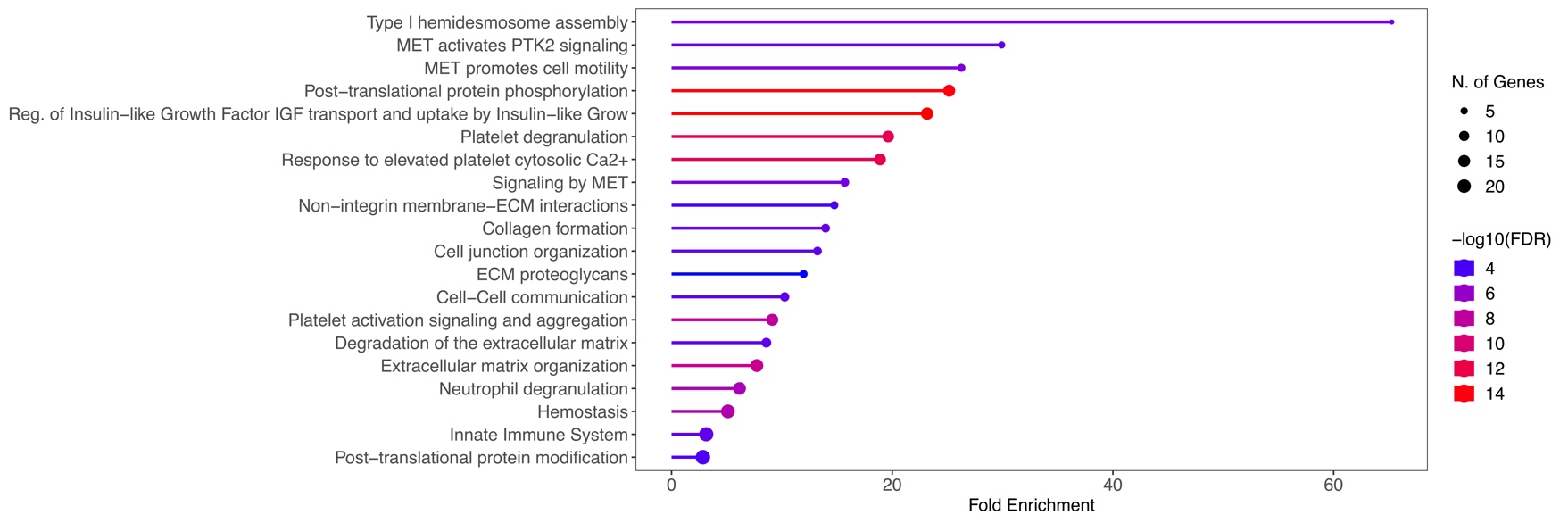


**Figure S8: Reactome pathway enrichment of proteins detected in individual EGFR mutant NSCLC cell lines vs HBEC GFP;p53^wt^.** Plot showing the top 20 pathways identified using the Curated.Reactome database (FDR < 0.05). Minimum pathway size was n = 2, maximum pathway size n = 2000.
